# Zero-Bias Photodetection and Opto-Synaptic Plasticity in BP/MoS_2_ and WS_2_/PdSe_2_ van der Waals Heterostructures

**DOI:** 10.1021/acsaelm.6c00115

**Published:** 2026-03-26

**Authors:** Ofelia Durante, Loredana Viscardi, Adolfo Mazzotti, Sebastiano De Stefano, Andres Castellanos-Gomez, Marika Schleberger, Antonio Di Bartolomeo

**Affiliations:** † Department of Physics “E. R. Caianiello”, 19028 University of Salerno, via Giovanni Paolo II, Fisciano, Salerno 84084, Italy; ‡ 2D Foundry Research Group, 69570 Instituto de Ciencia de Materiales de Madrid (ICMM-CSIC), Madrid E-28049, Spain; § 27170 Fakultät für Physik and CENIDE, Universität Duisburg-Essen, Lotharstrasse 1, Duisburg D-47057, Germany

**Keywords:** van der Waals heterostructures, black phosphorus, MoS_2_, WS_2_, PdSe_2_, mid-infrared photodetection, in-sensor memory, neuromorphic vision

## Abstract

van der Waals (vdW) heterostructures assemble atomically thin crystals into defect-free interfaces, where band alignment, interlayer coupling, and built-in electric fields can be engineered for multifunctional, low-power optoelectronics. This Spotlight provides a concise, critical overview of two complementary platforms - BP/MoS_2_ and WS_2_/PdSe_2_ - highlighting how their type-II or type-I band alignment enables optoelectronic operations. In back-gate transistors, both BP/MoS_2_ and WS_2_/PdSe_2_ heterojunctions exhibit transfer curves with high ON/OFF ratios that can reach 10^7^–10^8^ and hysteresis, which is reduced in a vacuum. In BP/MoS_2_, deterministic stacking and contact/work function engineering stabilize a type-II band offset that supports zero (*V*_ds_ = 0 V) or low (|*V*_ds_| ≤ 5 V) bias operation with fast, dual-time scale photoresponse (from hundreds of ms to ∼1 s) and linear behavior up to 50 μW optical power. Pressure tunes transport mechanisms (from thermionic to band-to-band tunnelling and ohmic), and four-probe data provide a band offset of 68 meV and zero-bias operation at 600 nm, consistent with MoS_2_-driven visible selectivity. Similarly, WS_2_/PdSe_2_ stacks enable visible-band photodetection at low bias, with pressure-tunable transport and photoresponse; the pressure-dependent photocurrent makes them promising for optoelectronic pressure sensing. In a gate-assisted regime (negative *V*_gs_ in high vacuum), persistent photoconductivity enables opto-synaptic plasticity in WS_2_/PdSe_2_ heterojunctions with paired-pulse facilitation (PPF) of 137% and post-tetanic potentiation (PTP) of 300% under 1 s pulse trains. Overall, this Spotlight examines synthesis pathways, interfacial structure, and device-level behavior, and discusses stability, uniformity, and readout compatibility as practical levers for translating single-device demonstrations into reproducible arrays.

## Graphical abstract

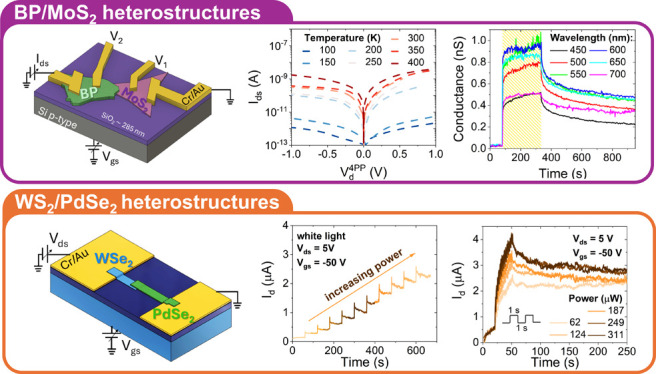

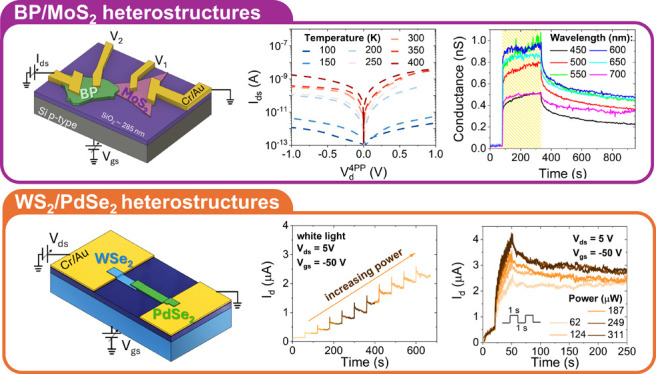

## 1 Introduction

Over the past five years, van der Waals (vdW) optoelectronics has progressed from proof-of-concept demonstrations to application-oriented device platforms.^1–18^ By assembling atomically thin crystals across clean interfaces, vdW heterostructures provide a modular route to optoelectronic functionalities that are difficult to achieve in bulk or epitaxial systems. Because adjacent layers couple through vdW forces rather than covalent bonds, lattice matching requirements are relaxed and interface disorder can be reduced. In this regime, device behavior is governed less by bulk composition and more by interface physics, specifically band alignment, interlayer coupling, and integrated fields, which can be designed to control carrier generation, separation, transport, and recombination with limited energy budgets. These ingredients have enabled the realization of pixels that operate at low (|*V*_ds_| ≤ 5 V) or zero (*V*_ds_ = 0 V) bias and begin to combine detection with elementary preprocessing or memory at the front end.^19,20^

This Spotlight focuses on two complementary vdW heterostructure families as case studies: black phosphorus/molybdenum disulfide (BP/MoS_2_) and tungsten disulfide/palladium diselenide (WS_2_/PdSe_2_). In BP/MoS_2_, the anisotropy and thickness-tunable bandgap of BP naturally pair with the robust semiconductor properties of MoS_2_, often producing type II offsets that favor charge separation and rapid photoresponsivity. In WS_2_/PdSe_2_, the strong optical transitions of WS_2_ combine with the electronic versatility of PdSe_2_ enabling alignment windows that include type I scenarios, paving the way for light-efficient recombination control and device functions beyond intensity sensing, such as multimodal operation and optoelectronic plasticity. Considered together, these platforms cover different areas of the design space and illustrate how interface control directly affects the behavior at the application level. The joint discussion of BP/MoS_2_ and WS_2_/PdSe_2_ is particularly instructive because the two systems cover complementary alignment regimes and functionalities. BP/MoS_2_ exemplifies type II, short-transit, low/zero-bias photodetection, while WS_2_/PdSe_2_ enables broadband operation and electrical reconfigurability. Together, they provide a design grammar that links synthesis and interfacial structure to device-level properties and functions ready for application in low-power multifunctional pixels. Against this backdrop, recent reports on BP/MoS_2_ and WS_2_/PdSe_2_ address complementary levers and, within this framework, establish practical design rules. As part of the “Spotlight on Applications” series, this article provides a concise and critical report on the current state of the art and summarizes representative findings into applicable design rules and trade-offs for low-power photodetection and sensor functionality. We use BP/MoS_2_ and WS_2_/PdSe_2_ as complementary case studies to link synthesis, assembly choices, and interfacial structure to operational regimes to practical constraints relevant to scalability.

In BP/MoS_2_, pressure-dependent transport in vertical junctions has been reported, with applied pressure acting as a post-fabrication knob to tune barrier heights and interlayer coupling while preserving low-power operation;^21^ self-powered photoconductivity with (near) zero bias and a stable, integrated, field-driven response suitable for programmable responsivity,^19^ and dominant n-type conduction with fast photoresponsivity, establishing contact/channel design rules for short-transition devices.^22^ In WS_2_/PdSe_2_, a visible-light photodetector with pressure-responsive behavior has been reported, highlighting the mechano-optical coupling in a single vdW junction;^23^ optoelectronic synaptic characteristics under optical/electrical pulse protocols have also been observed, indicating learning pathways in the sensor at the pixel level.^20^ To guide the reader, Table 1 summarizes the design space in a visual map linking platforms (BP/MoS_2_, WS_2_/PdSe_2_), controllable levers (device configuration, contacts/work function, pressure, gate), transport mechanisms (thermionic emission, band-to-band tunneling, persistent photoconductivity), and application metrics (*V*_oc_ at 0 V, ON/OFF, response time, PPF/PTP). This map is used as an organizing thread for the sections that follow.

**Table 1:** BP/MoS_2_ and WS_2_/PdSe_2_ Heterostructures: Key Parameters Taken from Selected Studies

ref	device configuration	spectra band	operating regime	key parameters
^1^	BP/MoS_2_ heterostructure; ultrafast pump–probe study	Visible (ultrafast excitation)	No device bias	Electron transfer ∼54 fs (1L-BP → MoS_2_); thickness-dependent charge transfer
^2^	BP/MoS_2_ phototransistor	Visible–NIR	Biased; wavelength/gate controlled	NPC ↔ PPC polarity switching; wavelength-tunable crossover (thickness/gate dependent)
^3^	MoS_2_/BP/MoS_2_ JFET (npn) photodetector	NIR → mid-IR (≈1550–3600 nm)	Low-bias JFET	*R* ≈ 9.04 A W^−1^ @ 1550 nm; D* ≈ 5.36 × 10^9^ Jones
^19^	Vertical 4-probe BP/MoS_2_ (self-powered)	Visible (∼600 nm)	0 V (photovoltaic)	Φ_B_ ≈ 68 meV (T-dependent *I*–*V*); zero-bias operation (mV-scale Voc)
^21^	Vertical BP/MoS_2_ heterojunction (back-gate)	Visible	Pressure-dependent transport	Thermionic → BTBT → ohmic regimes (kink at low V); dark current increases in high vacuum
^22^	BP/MoS_2_ junction with Cr asymmetric contacts	450–2400 nm (white-laser)	Biased (gate-tunable)	Dominant n-type conduction; fast photoresponse (≈sub-200 ms scale)
^16^	PdSe_2_/WS_2_ heterostructure photodetector (CVD WS_2_ + selenized Pd)	532–1550 nm	*V*_ds_ = 2 V (zero gate)	*R* = 3.91 mA W^−1^@ 635 nm; τ_rise/τ_decay = 49/90 ms
^17^	PdSe_2_/WS_2_ polarization-sensitive photosynapse	488–1550 nm	Synaptic operation; polarization readout	Polarization sensitivity ≈ 10.55 @ 980 nm; dark current ≈ 0.31 nA
^20^	WS_2_/PdSe_2_ photosynapse (opto-synaptic FET)	Visible–NIR	High vacuum; *V*_gs_ = –50 V	Persistent photoconductivity enabling synaptic plasticity under optical/electrical pulses; opto-synaptic plasticity with PPF ≈ 137% and PTP ≈ 300% under 1 s pulse trains. In-sensor (pixel-level) memory
^23^	WS_2_/PdSe_2_ heterostructure; back-gated visible photodetector and pressure sensor	Visible (peak @ 620 nm)	Low-bias (0–5 V); ambient ↔ 10^–4^ mbar	Responsivity up to 1.2 A W^–1^ @ 620 nm; pressure-tunable photocurrent (vacuum ↔ air)
^24^	WS_2_/PdSe_2_ heterostructure; three-terminal photoelectric synapse transistor	Visible	Gate-assisted synaptic operation; low-bias readout (*V*_ds_ = 1 V) with Vbg pulsed modulation; vacuum measurements	ON/OFF ≈ 10^6^; energy ≈ 2.4 pJ per event; multimodal analog synaptic plasticity and weight updateability; CIFAR-10 accuracy 92.8%

## 2 Results

In this section, this Spotlight further discusses the two selected platforms, BP/MoS_2_ and WS_2_/PdSe_2_, to illustrate how controlled synthesis and stacking determine the interfacial structure, which in turn governs device-level properties and, ultimately, application-ready functionalities. Together, these families occupy complementary alignment regimes (type II for BP/MoS_2_; tunable type I/II for WS_2_/PdSe_2_) and feature distinct knobs (contact/work function engineering, deterministic transfer, and atmosphere/pressure control) that translate interface physics into low-power pixels capable of sensing, storing, and performing elementary preprocessing on the sensor. The section is organized as follows: Section 2.1 discusses BP/MoS_2_ case studies spanning contacts/band alignment, pressure-tunable transport, and self-powered four-probe operation; Section 2.2 addresses WS_2_/PdSe_2_ junctions under pressure/adsorbate control and synaptic functionality enabled by persistent photoconductivity (PPC).

### 2.1 BP/MoS_2_ Heterostructures: Literature Survey

Recent literature underscores interface engineering as the primary lever governing carrier separation, transport, and functionality in BP/MoS_2_ heterostructures.

At the fundamental limit, pump–probe experiments report femtosecond interlayer electron transfer from single-layer BP to MoS_2_ within ∼ 54 fs - directly linking stacking geometry and layer thickness to exciton dissociation efficiency.^1^ Moving to device-level operation, both lateral and vertical BP/MoS_2_ junctions provide programmable polarity and band-edge selectivity as application levers that go beyond simple responsivity scaling.

In a representative BP-MoS_2_ phototransistor, photoconductance reversibly switches from negative to positive when excitation exceeds the MoS_2_ absorption limit; the crossover wavelength is set by MoS_2_ thickness and can be tuned electrostatically, enabling multistate logic and synaptic-like behaviors encoded in the photonic input. Switching occurs on practical time scales (seconds), illustrating how band alignment and trapping can be harnessed as functional resources rather than treated solely as limitations.^2^

Recent device reports further highlight how interfacial design extends performance into the near- and mid-infrared. A MoS_2_/BP/MoS_2_ junction FET photodetector achieves high sensitivity at telecommunications and mid-IR wavelengths (∼9.0 A W^–1^, D* ≈ 5.4 × 10^9^ Jones at 1550 nm; ∼7.3 A W^–1^, D* ≈ 4.3 × 10^9^ Jones at 3.6 μm), emphasizing efficient interlayer collection in a compact stack.^3^ BP/MoS_2_ functionalized heterojunctions also demonstrate how chemical engineering of the interface can switch between ultrafast sensing and nonvolatile optoelectronic memory: ON/OFF ratios ∼ 3.5 × 10^7^, dark current ∼0.13 pA, responsivity up to ∼22 A W^–1^, with rise/fall times ≈130/260 μs and ∼90 stable conductance states for in-sensor memory.^4^ Since the following figures of merit are extracted from different device architectures and operating conditions (e.g., illumination wavelength/irradiance, biasing, pressure/vacuum, and device area), they are reported here only to illustrate the overall qualitative performance and trade-offs relevant to the application, and are not intended for rigorous comparison between different studies.

Thermal transport across BP/MoS_2_ interfaces - critical for pixel stability under continuous-wave illumination - also benefits from vdW engineering: simulations indicate that interfacial thermal conductance can increase by ∼167% as temperature rises from 100 to 350 K, reflecting the sensitivity of phonon coupling to temperature and defect concentration.^5^ Collectively, complementary studies over the last five years further map a broad BP/MoS_2_ design space, including twist-/stacking-dependent electronic structure, gating-enabled nonlinear transport (e.g., Negative Differential Resistance), metasurface-assisted mid-IR photodiodes, and multifunctional heterostructure concepts, reinforcing interface control as a central lever for performance and versatility.^5–11,25–28^

Building on the BP/MoS_2_ literature context above, the following subsections highlight three representative vertical-stack case studies that operationalize complementary interface-control levers and enable practical take-home design rules. Section 2.1.1 addresses contact/band-alignment landscapes leading to dominant n-type conduction and fast photoresponse;^22^ Section 2.1.2 discusses pressure-dependent transport and the low-bias crossover from thermionic emission to band-to-band tunnelling;^21^ and Section 2.1.3 examines self-powered photoconduction in four-probe configurations to isolate junction-limited behavior and quantify the effective interfacial barrier.^19^

#### 2.1.1 Dominant n-Type Conduction with Fast Photoresponse

Recent work reports a BP/MoS_2_ heterostructure in which interface-controlled transport yields a fast photoconductive response characterized by two distinct time constants, linear power dependence, and pronounced bias-polarity asymmetry in the current arising from band alignment and contact effects.^22^ Atomic Force Microscopy (AFM) and Raman characterization (Figure 1a,b) support a clean heterogeneous interface with BP in the multilayer regime and MoS_2_ close to the monolayer limit, while time-resolved photocurrent traces (Figure 1c-d) reveal a fast component (τ_1_, hundreds of ms) and a slower component (τ_2_, hundreds of ms to ∼1 s), commonly associated with the presence of BP in heterostructures, characterized by higher mobility than MoS_2_, and slower trapping/detrapping at the interface and flake edges.

**Figure 1:**
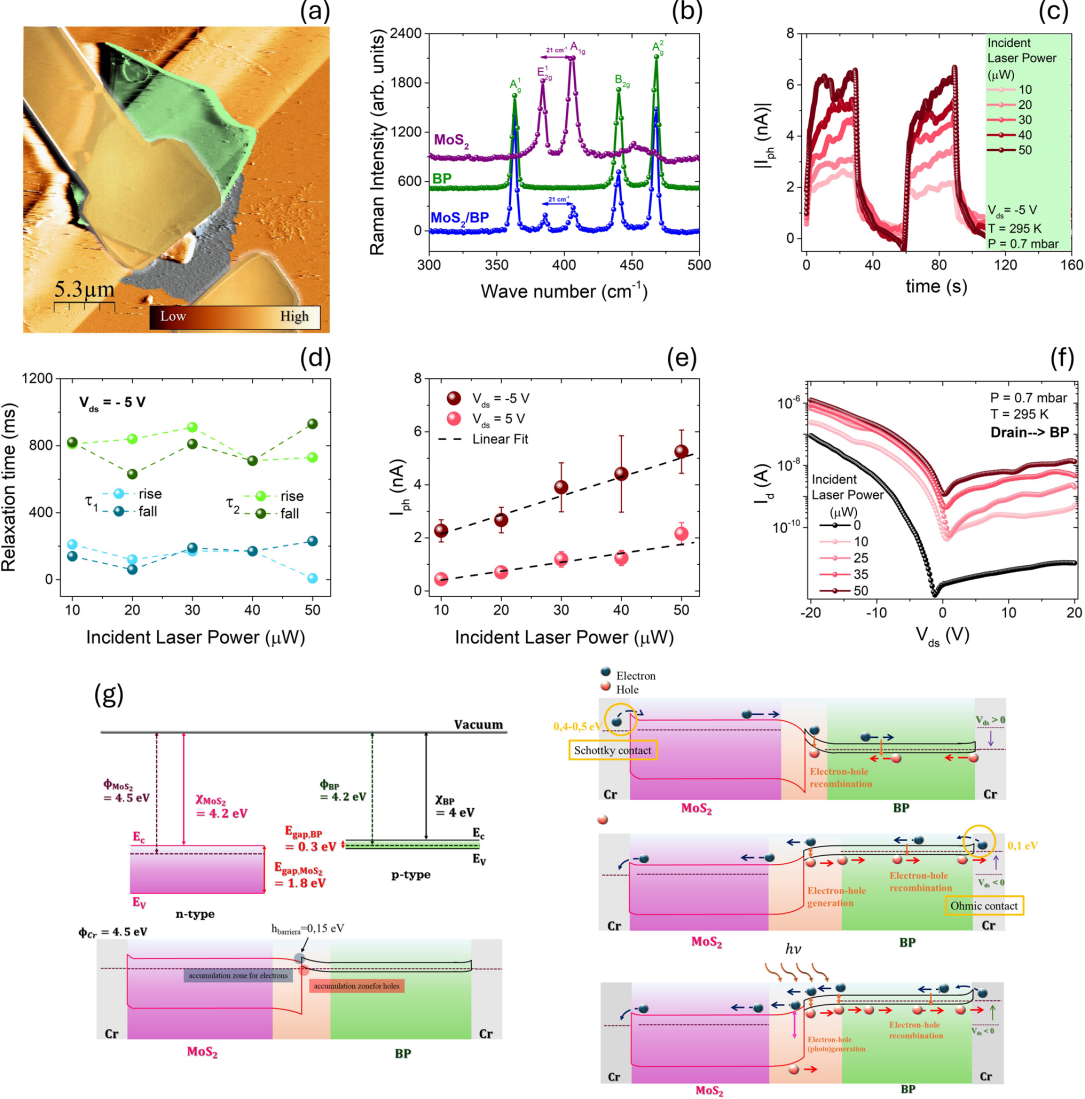
(a) AFM composite of the BP/MoS_2_ heterojunction with Cr/Au electrodes. (b) Raman spectra of pristine MoS_2_ and BP and of the overlap region (BP/MoS_2_). (c) Time-resolved photocurrent traces under white-laser illumination for increasing incident power (10–50 μW) at *V*_ds_= −5 V (295 K, 0.7 mbar). (d) Rise/decay constants versus incident power extracted from double-exponential fits. (e) Photocurrent versus incident power at *V*_ds_= ± 5V (linear regime over the tested range). (f) Output characteristics in dark and under illumination (drain connected to BP; 0.7 mbar, 295 K). (g) Band-diagram sketches of BP/MoS_2_ with Cr contacts at equilibrium and under bias/illumination, highlighting a type-II offset, Schottky contact at Cr/MoS_2_ (electron barrier ≈ 0.4–0.5 eV) and ohmic contact at Cr/BP, which together rationalize the dominant n-type transport and the larger photocurrent for negative *V*_ds_. Adapted from ref 22. Copyright 2024 The Author(s). Published by Elsevier B.V. under the CC BY 4.0 license.

Power-dependent measurements (Figure 1e) show a linear scaling of *I*_ph_ with incident power at *V*_ds_ = ± 5 V, supporting a photoconductive mechanism where free-carrier density increases with photon flux. The polarity asymmetry of the current with higher current at negative *V*_ds_ (Figure 1f) can be rationalized by a staggered type-II junction predicted by Anderson’s rule, together with asymmetric Cr contacts (ohmic type at interface with BP and Schottky type with 0.4–0.5 eV barrier on MoS_2_).

Using representative band parameters (MoS_2_: Φ ≈ 4.5 eV, χ ≈ 4.2 eV, *E*_g_ ≈ 1.8 eV;^9,29,29,30^ BP: Φ ≈ 4.2 eV, χ ≈ 4.0 eV, *E*_g_ ≈ 0.3 eV^9,31,32^ ), the BP/MoS_2_ interface is consistent with a modest electron barrier (∼0.15 eV) that only weakly impedes transport. Indeed, as demonstrated in the following, four-probe measurements indicate a slightly lower barrier of 0.068 eV. The band bending suppresses hole transport from BP to MoS_2_ while enabling electron transport, although electron transfer from MoS_2_ to BP remains limited by the Cr/MoS_2_ Schottky barrier. The band alignment enables efficient photocarrier separation, particularly under reverse bias conditions (*V*_ds_ < 0 V), with electrons drifting toward the MoS_2_ layer and holes toward the BP layer (Figure 1g).

In BP/MoS_2_ vertical stacks, a type-II offset combined with asymmetric contacts can yield polarity-selective, linear photoconductive gain; however, maintaining fast, reproducible response requires suppressing interfacial trapping.

#### 2.1.2 Pressure-Dependent Transport in Vertical Stacks

Pressure provides a practical knob to modulate transport in vertical BP/MoS_2_ heterojunctions. Despite identical Cr/Au contacts, the polarity asymmetry shown in Figure 2a,b differs from the electrical measurements reported in Figure 1f, indicating that junction electrostatics dominate over metal choice alone. In particular, differences in BP thickness can modify gate screening and series resistance, shifting where voltage drops and how band bending develops across the overlap region.

**Figure 2:**
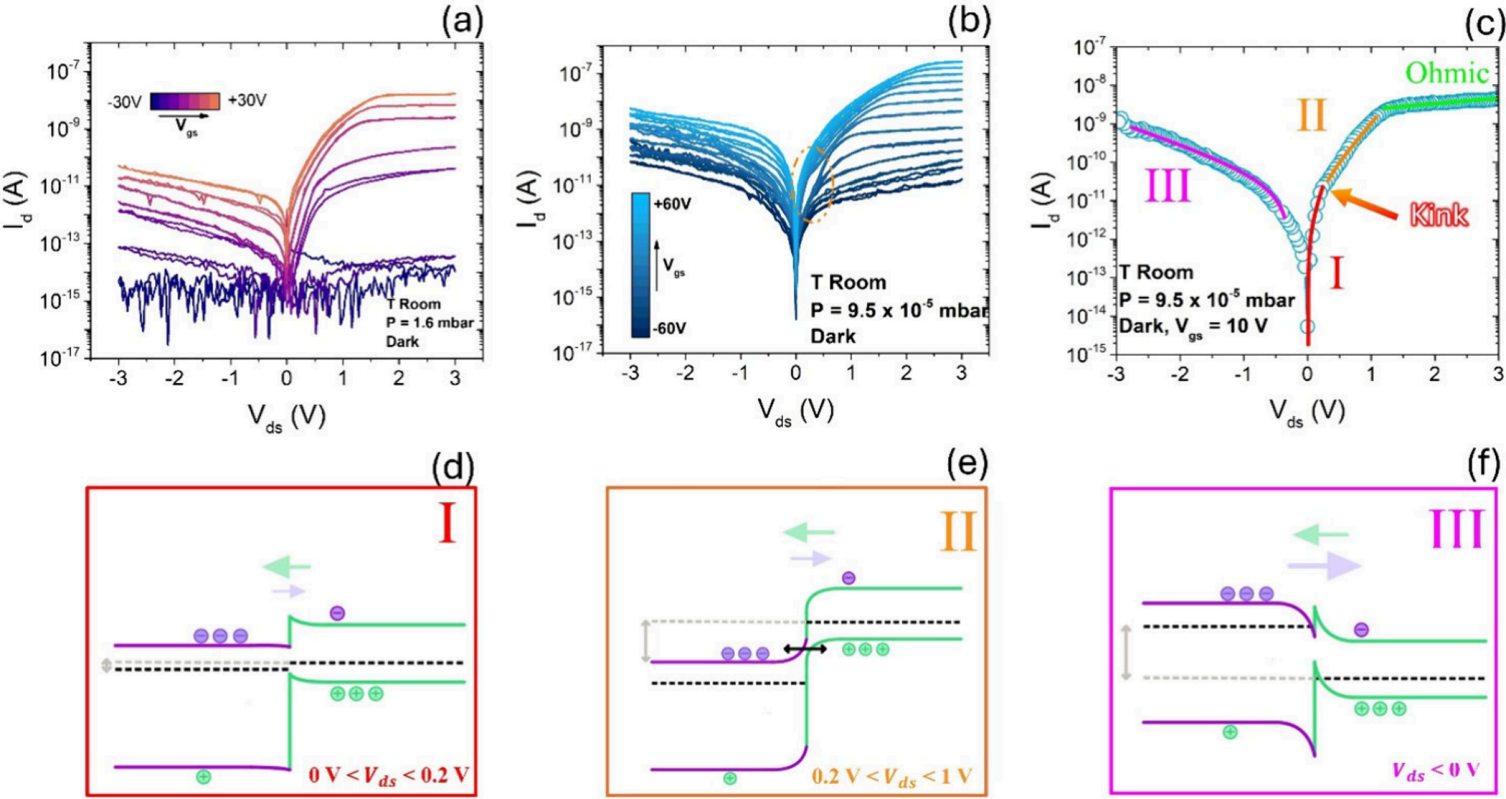
Dark output characteristics at (a) 1.6 mbar and at (b) 9.5 × 10^–5^ mbar for different *V*_gs_. Panel (b) shows bias-polarity asymmetry and “kink” in the current at small bias. (c) Dark output curve at *V*_gs_ = +10 V measured at 9.5 × 10^–5^ mbar. Higher vacuum yields larger dark current and a pronounced kink. The fitting lines indicate dominant transport mechanisms: thermionic emission, band-to-band tunnelling (BTBT), and ohmic transport. (d-f) Type-II BP/MoS_2_band diagrams illustrating three conduction regimes: **I** (0 < *V*_ds_ < 0.2 V) thermionic emission from BP to MoS_2_; **II** (0.2 < *V*_ds_ < 1 V) BTBT across the junction; **III** (*V*_ds_ < 0) current limited by the MoS_2_/metal Schottky barrier and the BP/MoS_2_ interfacial barrier, consistent with n-type behavior. Reproduced with permission from ref 21. Copyright 2025 The Author(s). Published by Elsevier Ltd. Licensed under CC BY-NC-ND 4.0.

Under reduced pressure, suppression of adsorbate-induced trapping increases the dark current in BP/MoS_2_ heterojunctions with Schottky contacts and reveals a characteristic low-bias “kink” in forward *I*–*V* curves, marking a crossover from thermionic emission to band-to-band tunnelling (BTBT).^21^ As shown in Figure 2a, the dark output characteristics at 1.6 mbar are lower than those at 9.5 × 10^–5^ mbar (Figure 2b), consistent with reduced adsorption and a decreased trap/scattering density under high vacuum conditions. The “kink”, highlighted in Figure 2b by an orange dotted circle, appears around *V*_*ds*_ ≈ 0.1 V for positive *V*_gs_ and can be interpreted in terms of a small set of transport regimes (Figure 2c): (i) at low forward bias (0–0.2 V), Figure 2d, Schottky-like thermionic emission dominates; (ii) at intermediate bias (0.2–1 V), Figure 2e, BTBT becomes accessible and produces the kink/changed slope; and (iii) at higher bias (*V*_ds_ > 1 V), series resistance in the BP and MoS_2_ channels drives an effectively ohmic response. For *V*_ds_ < 0, Figure 2f, transport is strongly suppressed and limited by the MoS_2_/metal Schottky barrier together with the BP/MoS_2_ interfacial barrier, consistent with the overall n-type character of the stack. Pressure - via adsorbate/trap control - remodels the effective barrier landscape and can change the dominant transport pathway (thermionic → BTBT), enabling behavior similar to that of a low-voltage, high-gain tunnel diode under high vacuum conditions or low-noise regimes at higher pressures relevant to detection modes.

#### 2.1.3 Self-Powered Photoconduction

Self-powered operation in BP/MoS_2_ junctions is better assessed using a four-point contact (4PP) geometry (two electrodes per flake), which minimizes contact-resistance artifacts and highlights junction-related transport. In the configuration described in ref 19, photovoltaic parameters (short circuit current, *I*_sc_, and open circuit voltage, *V*_oc_) are evaluated versus temperature (100–400 K) and wavelength (450–700 nm), clarifying how the effective interfacial barrier and spectral absorption govern the photoresponse. Device architecture and optical characterization are shown in Figure 3a,b, while dark *I*–*V* curves acquired from 100 to 400 K under reduced pressure (Figure 3c) show increasing conductance and evolving rectification with temperature, consistent with thermally activated release from traps/defects and progressive desorption of O_2_/H_2_O that reduces scattering and increases electron density. A standard thermionic-emission diode analysis^33^ yields a small effective interfacial barrier for electrons, Φ_B_ ∼ 68 meV at *V*_ds_ = −1 V (Richardson plot inset of Figure 3c), indicating a weakly electron-blocking interface compatible with what was reported before.^34^ Under monochromatic illumination (450–700 nm), 4PP I–V curves retain rectification and exhibit a clear photovoltaic response at zero (V_ds_ = 0 V) bias (Figure 3d), with *I*_sc_ and *V*_oc_ varying with wavelength. *V*_oc_ reaches ∼2.5 mV at λ ≈ 600 nm (inset of Figure 3d), and time-resolved measurements (Figure 3e,f) show that photoconductance peaks for λ ≲ 600 nm and decreases for longer wavelengths, consistent with efficient excitation when photon energy exceeds the MoS_2_ bandgap (∼1.8 eV).

**Figure 3:**
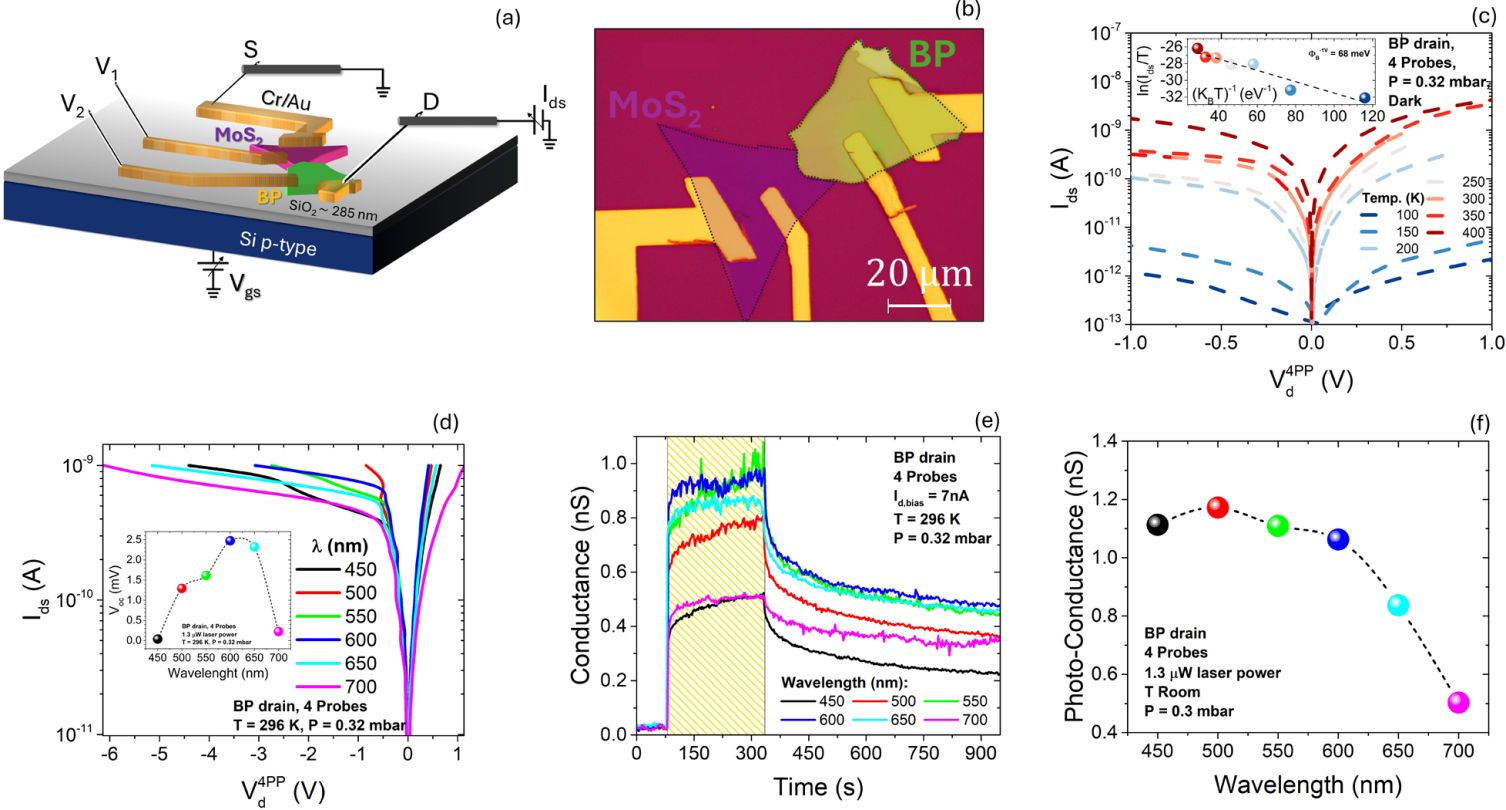
(a) Schematic of the BP/MoS_2_ heterostructure with Cr/Au leads in a four-probe configuration. (b) Processed 100× optical image highlighting the overlap region. (c) Four-probe *I*–*V* characteristics of the junction in the dark at different temperatures; inset: Richardson plot at *V*_ds_ = −1 V, and linear fit (dashed curves) with a dashed linear fit used to extract the barrier height, Φ_B_. (d) Four-probe *I*–*V* curves under monochromatic illumination at selected wavelengths; inset: Open circuit voltage as a function of the wavelength. (e) Conductance versus time at fixed drain current *I*_d_ = 7 nA, during exposure to a single laser pulse with wavelength stepped from 400 to 700 nm. (f) Photoconductance as a function of the wavelength. Adapted from ref 19. Copyright 2025 The Author(s). Published by Wiley-VCH GmbH under the CC BY 4.0 license.

Light-dark transitions again display two characteristic time constants, attributable to fast channel transport and slower trapping/detrapping at the interface. Four-probe measurements show that a small effective interfacial barrier enables self-powered MoS_2_/BP operation with visible-band selectivity (peak near ∼600 nm). Engineering Φ_B_ and trap dynamics is therefore a direct route to stable, ultralow-power front-end pixels and spectrally selective sensing.

### 2.2 WS_2_/PdSe_2_ heterostructures: Literature survey

The WS_2_/PdSe_2_ material pair offers a complementary vdW platform in which broadband optical absorption combines with electrical reconfigurability driven by band alignment, defect chemistry, and environmental coupling. WS_2_ supplies robust excitonic transitions and controllable doping, whereas PdSe_2_ contributes a narrow-gap electronic channel with strong sensitivity to adsorbates and gate fields. At the device level, WS_2_/PdSe_2_ vdW photodiodes allow control on junction polarity and rectification: gate-tunable switching between p-n and n-p modes and rectification ratios continuously adjustable from ∼10^–4^ to ∼10^4^ via band-to-band tunnelling control have been reported, which is attractive for polarity-programmable front ends.^12^

The key properties of WS_2_ - carrier mobility, excitonic optical constants, and defect chemistry - are now well mapped, providing a mature toolbox for doping/defect engineering and synthesis routes that translate directly into heterostructure performance.^35^

Beyond visible-band operation, WS_2_ -based broadband stacks highlight strategies for zero-bias and infrared response through defect-assisted gap reduction and optical field management. A pyramidal WS_2_/Si mixed junction achieves *R* ≈ 0.29 A W^–1^, D* ≈ 2.6 × 10^14^ Jones, and ultrabroad response spectrum ranging from 265 nm to 3.0 μm at 0 V,^13^ while WS_2_/Si type-II junctions report responsivity of ∼23 A W^–1^and specific detectivity ∼1.6 × 10^12^ Jones with mid-IR response, indicating viable building blocks for low-power arrays.^14^ Directional/polarization-sensitive functionality can also be introduced by coupling WS_2_ with anisotropic layers: WS_2_/BP stacks retain robust anisotropy in optical response (e.g.,∼1.8× polarization ratio for neutral excitons), pointing to direction-sensitive pixels without external polarizers.^15^ The same materials set further supports cross-domain sensing modalities through complementary surface chemistry.

PdSe_2_ films anchored to WS_2_ yield a reported ∼67% response to 50 ppm of H_2_ at 100 °C, illustrating cointegrated environmental sensing based on chemo resistive/catalytic effects.^36^ Additional photodiode implementations reinforce broadband detection capabilities in WS_2_/PdSe_2_: a device fabricated via direct selenization of Pd onto WS_2_ monolayers followed by transfer is reported to exhibit type-I band alignment and a broadband response from 532 to 1550 nm.^16^ Under 635 nm excitation, rise/fall times of ∼49/90 ms at *V*_ds_ = 2 V and a sublinear photocurrent-intensity scaling (α ≈ 0.76–0.85) are consistent with trap-assisted recombination and lifetime limitations; ON/OFF reaches ∼10^3^ in ambient conditions, and responsivity peaks at low irradiance (e.g., ∼3.9 mA W^–1^) before decreasing at higher power.^16^

Synaptic-type operation has been demonstrated in a two-terminal WS_2_/PdSe_2_ photosynapse spanning the visible-to-infrared (≈488–1550 nm), combining very low dark current (∼0.31 nA) with strong bias sensitivity (ratio ≈10.55 at 980 nm) and device-level learning functions (e.g., paired pulse facilitation PPF ≈138% and bias-encoded optical programming/reading of synaptic weights).^17^ Moreover, three-terminal 2D WS_2_/PdSe_2_ can reach an ON/OFF ratio of ∼10^6^ operating at an ultralow energy cost of ∼2.4 pJ per event. Under electrical gating, the device supports multimodal analog synaptic functions, including robust plasticity and reliable, updateable synaptic weights.^24^

Building on the broader WS_2_/PdSe_2_ landscape outlined above, the following subsections highlight two representative case studies that leverage the strong environmental coupling of this interface in complementary ways. Section 2.2.1 focuses on pressure/adsorbate-controlled transport and photoresponse to realize an optoelectronic pressure sensor, while Section 2.2.2 discusses persistent-photoconductivity regimes under vacuum and gate bias that enable synapse-like optoelectronic plasticity.

#### 2.2.1 WS_2_/PdSe_2_ Heterostructures as a Pressure Optoelectronic Sensor

WS_2_/PdSe_2_ stacks provide an optoelectronic pressure-sensing modality in which ambient conditions tune both transport and photoresponse. Varying pressure from 10^–4^ mbar to ambient under controlled illumination produces reversible shifts in transfer characteristics and a photocurrent that is strongly pressure dependent. Devices assembled by mechanical exfoliation and deterministic dry transfer,^37^ (Figure 4a) offer clean interfaces suitable for isolating adsorbate-driven effects; AFM thickness estimates (Figure 4b) place WS_2_ close to the monolayer regime. Figure 4c illustrates the WS_2_/PdSe_2_ band alignment before (top) and after (bottom) contact. In the top panel, isolated materials are referenced to the vacuum level using ϕ, χ, and *E*_gap_, yielding the positions of *E*_C_, *E*_V_, and *E*_F_; WS_2_ shows a wide gap (∼2 eV)^35^ and n-type behavior, whereas PdSe_2_ has a small gap (∼0.1 eV)^38^ and p-type character with *E*_F_ near *E*_V_. The other parameters for WS_2_ are ϕ = 4.6 eV, χ = 3.9 eV, and for PdSe_2_ they are ϕ = 5.1 eV, χ = 4.6 eV. In the bottom panel, when contact is established, Fermi-level equilibration drives electron transfer from WS_2_ to PdSe_2_ (ϕ_WS2_ < ϕ_PdSe2_), depleting WS_2_ and partially compensating holes in PdSe_2_ near the interface. The resulting built-in field sets a type-I alignment that favors visible-band absorption in WS_2_ and charge storage in PdSe_2_. Figures 4d,e confirm that WS_2_ is n-type with mobility that increases markedly in high vacuum, while PdSe_2_ evolves from ambipolar behavior at ambient pressure to predominantly n-type after removal of H_2_O/O_2_ adsorbates. In the heterostructure, these adsorbate-controlled polarity and mobility shifts translate into pressure-tunable transfer curves (Figure 4f): hysteresis narrows from ∼70 V at ambient to ∼15 V in high vacuum, consistent with reduced charge trapping upon desorption, and strong gate control is retained with ON/OFF ∼10^7^–10^8^ across the pressure cycle.

**Figure 4:**
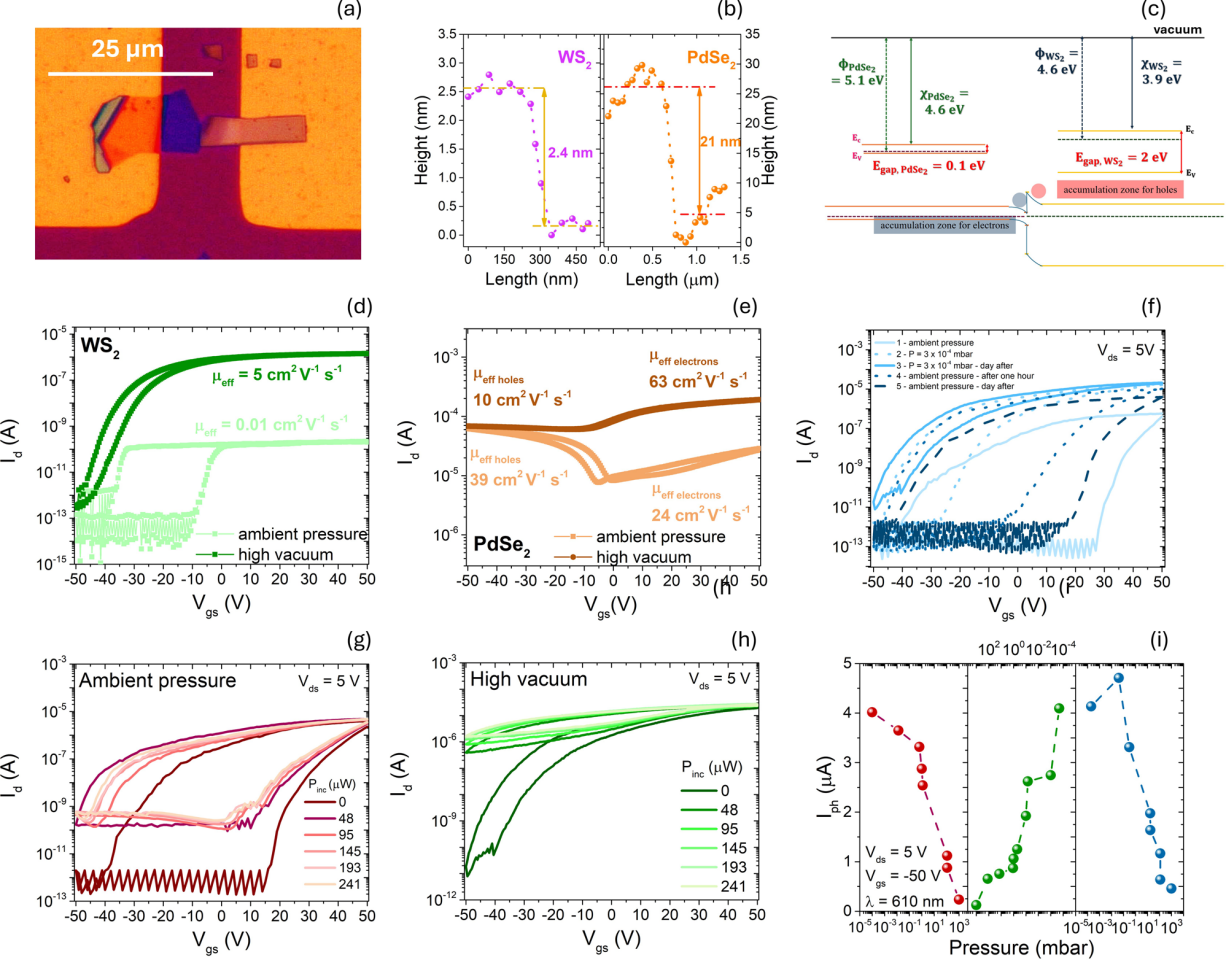
(a) Optical micrograph of a WS_2_/PdSe_2_ heterostructure. (b) AFM height line profiles for the flakes: WS_2_ (left) and PdSe_2_ (right). (c) WS_2_/PdSe_2_ band alignment before (top) and after (bottom) contact. Work function and electron affinity values are representative and may vary with thickness, doping, and adsorbates (environment).^39,40^ Transfer curves at ambient pressure and in high vacuum at a pressure of 10^–4^ mbar for a single (d) WS_2_ and (e) PdSe_2_ flake-based devices. (f) Transfer curves of the WS_2_/PdSe_2_ heterostructure at a fixed *V*_ds_ = 5 V in different states. Transfer curves of the WS_2_/PdSe_2_ heterostructure under supercontinuum laser at (g) ambient pressure and in (h) high vacuum at a pressure of 10^–4^ mbar. (i) Photocurrent of the WS_2_/PdSe_2_ heterostructure at λ = 620 nm under pressures from high vacuum at 10^–4^ mbar to ambient pressure. Adapted from ref 23. Copyright 2025 The Author(s). Published by IOP Publishing Ltd., under the CC BY 4.0 license.

Under white-light illumination (Figures 4g,h), current increases at all gate biases and the transfer curve shifts more strongly in high vacuum, preventing complete turn-off within the measured *V*_gs_ window. Spectral measurements further show a pressure-dependent photocurrent at λ = 620 nm (Figure 4i), directly linking adsorbate state to optoelectronic output. Overall, the combined pressure-tunable transfer characteristics and visible-band photoresponse support WS_2_/PdSe_2_ heterostructures as candidates for low-power on-chip phototransistors and for chemical/environmental sensing modalities where adsorbates act as functional knobs. In WS_2_/PdSe_2_, adsorbate-controlled polarity/mobility and trap-mediated hysteresis provide an intrinsic route to pressure-tunable electronics and photoresponse, enabling multimodal (light + pressure) sensing at low bias.

#### 2.2.2 WS_2_/PdSe_2_ Heterostructures as an Optoelectronic Synaptic Device

WS_2_/PdSe_2_ junctions can exhibit persistent photoconductivity (PPC) under high vacuum and negative gate bias, enabling synapse-like optoelectronic behavior under optical/electrical pulse stimuli. Devices assembled by deterministic stacking on a back-gated Si/SiO_2_ platform (Figure 5a)^37^ show PPC-driven conductance states consistent with a history-dependent “synaptic weight”. Power-dependent measurements (Figure 5b) show that illumination increases the drain current and that successive pulses incrementally raise the conductance, while the conductance remains elevated after the light is switched off. Under negative gate bias (e.g., *V*_gs_ = −50 V), trapped holes at interfacial sites suppress recombination and prolong electron lifetime in the conduction channel, providing a physical basis for PPC and pulse-programmable conductance.

**Figure 5:**
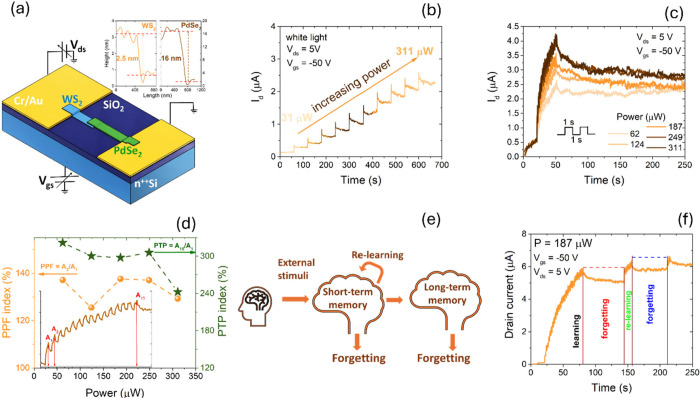
(a) Schematic of the device and electrical setup; inset: AFM line profiles of both flakes. (b) Response to individual 1-s light pulses at *V*_ds_ = 5 V and *V*_gs_ = −50 V as the incident laser power increases. (c) Train of 15 1-s pulses separated by 1 s at *V*_ds_ = 5 V and *V*_gs_ = −50 V at different incident laser power. (d) PPF (left axis) and PTP (right axis) versus incident laser power. (e) Conceptual diagram of brain operation. (f) Demonstration of learning → forgetting → relearning in the WS_2_/PdSe_2_ synaptic device. Adapted from ref 20. Copyright 2025 The Author(s). Published by IOP Publishing Ltd., under the CC BY 4.0 license.

In this regime, pulse trains produce stepwise current increments (Figure 5c). Paired-pulse facilitation (PPF) is quantified as *A*_2_/*A*_1_ (in %), where *A*_1_and *A*_2_ are the peak current responses to two consecutive light pulses, yielding PPF ≈ 137%.^41,42^ Post-tetanic potentiation (PTP) (in %) is quantified as *A*_n_/*A*_1_ (in %), where *A*_n_ is the peak response after a pulse train; PTP remains around ≈ 300% (Figure 5d).^43^ Learning-forgetting-relearning cycles are also reproduced (Figures 5e,f): a training sequence increases conductance (learning), followed by gradual decay (forgetting), and subsequent stimuli recover the trained state with fewer pulses (relearning), consistent with accelerated recall relative to initial training. PPC under high vacuum and negative gate bias provides a programmable, history-dependent conductance state in WS_2_/PdSe_2_ that can encode synaptic weight (PPF/PTP and learning cycles); array-level stability and reproducibility will hinge on controlling traps and environmental coupling.

## 3 Conclusions and Outlook

This Spotlight connects deterministic assembly and interfacial design to device-level metrics and application-oriented behavior in two complementary vdW platforms: BP/MoS_2_ and WS_2_/PdSe_2_. In this article, we provide a concise and critical status report and extract actionable design rules linking synthesis and assembly choices and interfacial structure to operational regimes and integration constraints relevant to scalable low-power pixels. Clean, completely dry transfers, controlled alignment of thickness/twist bands, and trap landscapes that regulate generation, separation, transport, and recombination with limited energy budgets emerge as recurring enablers for front-end pixels capable of beginning to detect, store, and preprocess information. In vertical layouts, BP/MoS_2_ acts as a short-passage type II archetype. Recent work reports n-type conduction with fast and reproducible photoresponsivity, showing an almost linear scaling with incident power and a clear polarity asymmetry that is consistent with interfacial band offsets and asymmetric Cr contacts. Complementary measurements across ambient-to-high-vacuum conditions reveal a low-bias “kink” associated with a crossover from thermionic emission to band-to-band tunnelling, indicating pressure as a practical post-fabrication lever for adjusting barriers and coupling between layers. Four-probe measurements further support self-powered photoconduction with a small interfacial barrier (∼68 meV), visible-band selectivity with a peak near ∼600 nm, and two characteristic time scales related to fast channel transport and slower trapping/detrapping dynamics. Collectively, these studies point to practical design rules for engineering contacts and interfaces in vertical, short-transit photodetectors operating at zero (*V*_ds_ = 0 V) and low bias (|*V*_ds_| ≤ 5 V).

The WS_2_/PdSe_2_ platform complements the BP/MoS_2_ heterojunction and enables broadband absorption together with reconfigurability. Single flake controls showed pressure-induced mobility/polarity changes which, when combined in a stack, produce tunable gate phototransistors with large ON/OFF ratios (10^7^–10^8^), reduced hysteresis under high vacuum, and a pressure-coupled photoresponse pathway toward multimodal pixels. In a gate-assisted regime (negative V_gs_ in high vacuum), the junction exhibits persistent photoconductivity enabling optoelectronic synaptic behavior: short-term plasticity with PPF ≈ 137% and PTP ≈ 300% and learning-forgetting-relearning similar to those in the brain, where fewer pulses are needed to recover a trained state. Taken together, these observations support the existence of internal memory within the sensor and allow for preprocessing directly at the pixel level, enabling novel applications in unconventional computing.

The translation of the performance of a single device into reproducible arrays appears to hinge on a small set of immediate measures:

(i) Stability and passivation, particularly for BP (e.g., h-BN/ALD encapsulation) to curb oxidation and drift;
(ii) Uniformity/variability control via deterministic stacking, twist/thickness metrology (±1–2°; ±1 layer) and gentle modeling of the trap landscape;
(iii) Short-transit vertical architectures that support zero/low bias operation without sacrificing bandwidth;
(iv) Readout compatibility, CDS/noise budgeting, and per-pixel programmability of bias response and synaptic weight within a CMOS back-end. As an illustrative intermediate reference, a modestly sized array (e.g., 32 × 32) that achieves high functional efficiency (on the order of ≳95%) and controlled pixel-to-pixel sensitivity dispersion (on the order of ∼10–15%) would represent a useful step forward compared to individual devices; however, the required array size and acceptable dispersion depend on the application and architecture and can be mitigated by calibration and readout/processing schemes.

More broadly, the convergence of interface control and programmable junction physics enables pixels that detect, store, and preprocess information at extremely low power: from zero-bias BP/MoS_2_ photodiodes delivering stable front-end signals, to WS_2_/PdSe_2_ elements that embed synaptic weights and multimodal (light + pressure) signaling in hardware. Looking ahead, heterogeneous focal planes combining BP/MoS_2_ for NIR/MWIR detection with WS_2_/PdSe_2_ for visible/polarimetry/learning could shift computation toward the sensor, reduce data movement, and support efficient neuromorphic vision. With continued advances in passivation, uniformity, and vertical transport, vdW heterostructures are poised to move from interesting device concepts to scalable, ready-to-use arrays for robust imaging and low-power on-pixel intelligence.

## References

[1] Yin Y., Zhao X., Ren X., Liu K., Zhao J., Zhang L., Li S. (2022). Thickness Dependent Ultrafast Charge
Transfer in BP/MoS_2_ Heterostructure. Adv. Funct Materials.

[2] Jawa H., Varghese A., Ghosh S., Sahoo S., Yin Y., Medhekar N. V., Lodha S. (2022). Wavelength-Controlled
Photocurrent
Polarity Switching in BP-MoS_2_ Heterostructure. Adv. Funct Materials.

[3] Shu X., Wu J., Zhong F., Zhang X., Fu Q., Han X., Zhang J., Lu J., Ni Z. (2024). High-Responsivity,
High-Detectivity, Broadband Infrared Photodetector Based on MoS2/BP/MoS2
Junction Field-Effect Transistor. Appl. Phys.
Lett..

[4] Liu C., Ding S., Tian Q., Hong X., Su W., Tang L., Wang L., Zhang M., Liu X., Lv Y., Ho J. C., Liao L., Zou X. (2023). Realizing the Switching
of Optoelectronic Memory and Ultrafast Detector in Functionalized-Black
Phosphorus/MoS _2_ Heterojunction. Laser &amp; Photonics Reviews.

[5] Wu B., Zhou M., Xu D., Liu J., Tang R., Zhang P. (2022). Interfacial Thermal Conductance of
BP/MoS2 van Der Waals Heterostructures:
An Insight from the Phonon Transport. Surfaces
and Interfaces.

[6] Wei D., Li Y., Guo G., Yu H., Ma Y., Tang Y., Feng Z., Dai X. (2024). Tunable Electronic
and Optical Properties
of H-BP/MoS2 van Der Waals Heterostructures toward Optoelectronic
Applications. J. Phys. Chem. Solids.

[7] Wu F., Tian H., Yan Z., Ren J., Hirtz T., Gou G., Shen Y., Yang Y., Ren T.-L. (2021). Gate-Tunable Negative
Differential Resistance Behaviors in a hBN-Encapsulated BP-MoS _2_ Heterojunction. ACS Appl. Mater. Interfaces.

[8] Joseph I., Wan K., Hussain S., Guo L., Xie L., Shi X. (2021). Interlayer
Angle-Dependent Electronic Structure and Optoelectronic Properties
of BP-MoS2 Heterostructure: A First Principle Study. Comput. Mater. Sci..

[9] Jiang X., Zhang M., Liu L., Shi X., Yang Y., Zhang K., Zhu H., Chen L., Liu X., Sun Q., Zhang D. W. (2020). Multifunctional Black Phosphorus/MoS _2_ van
Der Waals Heterojunction. Nanophotonics.

[10] Lien M. R., Wang N., Guadagnini S., Wu J., Soibel A., Gunapala S. D., Wang H., Povinelli M. L. (2023). Black Phosphorus
Molybdenum Disulfide Midwave Infrared Photodiodes with Broadband Absorption-Increasing
Metasurfaces. Nano Lett..

[11] Raturi M., Kaur A., Tyagi H., Bhakar M., Saini J., Kaur M., Sarkar A. D., Hazra K. S. (2023). Nanoscale Probing
of Surface Potential Landscape at MoS_2_ /BP van Der Waals
p–n Heterojunction. Nanotechnology.

[12] Chen T., Wu Q., Gao Y., Wang J., Wang X., Wang X., Yan S., Shi Y. (2024). Reconfigurable Single-Gate PdSe2/WS2 Diode with High
Symmetry Rectification. Sci. China Mater..

[13] Wu D., Guo C., Wang Z., Ren X., Tian Y., Shi Z., Lin P., Tian Y., Chen Y., Li X. (2021). A Defect-Induced Broadband
Photodetector Based on WS_2_ /Pyramid Si 2D/3D Mixed-Dimensional
Heterojunction with a Light Confinement Effect. Nanoscale.

[14] Wu E., Wu D., Jia C., Wang Y., Yuan H., Zeng L., Xu T., Shi Z., Tian Y., Li X. (2019). In Situ Fabrication
of 2D WS_2_ /Si Type-II Heterojunction for Self-Powered Broadband
Photodetector with Response up to Mid-Infrared. ACS Photonics.

[15] Li X., Xie X., Wu B., Chen J., Li S., He J., Liu Z., Wang J.-T., Liu Y. (2024). Observation of Robust
Anisotropy
in WS2/BP Heterostructures. Nano Res..

[16] Kang X., Lan C., Li F., Wang W., Yip S., Meng Y., Wang F., Lai Z., Liu C., Ho J. C. (2021). Van Der
Waals PdSe_2_ /WS_2_ Heterostructures for Robust
High-Performance Broadband Photodetection from Visible to Infrared
Optical Communication Band. Advanced Optical
Materials.

[17] Fan W., Yan H., Wang X., Tong L., Yan W., Su C., Wang Q., Yin S. (2025). Polarization-Sensitive Photosynapse
Based on PdSe_2_ /WS_2_ Heterostructure for Visible-Infrared
Broadband Artificial Vision System. Adv. Funct
Materials.

[18] Wu F., Zhu Z.-Q., Tian H., Yan Z., Liu Y., Xu Y., Xing C.-Y., Ren T. (2022). Vertical WSe2/BP/MoS2 Heterostructures
with Tunneling Behaviors and Photodetection. Appl. Phys. Lett..

[19] Mazzotti A., Durante O., De Stefano S., Viscardi L., Pelella A., Kharsah O., Daniel L., Sleziona S., Schleberger M., Di Bartolomeo A. (2025). BP/MoS_2_ Van Der Waals Heterojunctions for
Self-Powered Photoconduction. Advanced Optical
Materials.

[20] Viscardi L., Mazzotti A., Durante O., Pucher T., Martucciello N., Castellanos-Gomez A., Bartolomeo A. D. (2025). Optoelectronic Synaptic Characteristics
of a van Der Waals WS_2_ /PdSe_2_ Heterostructure. J. Phys. D: Appl. Phys..

[21] Durante O., De Stefano S., Mazzotti A., Viscardi L., Giubileo F., Kharsah O., Daniel L., Sleziona S., Schleberger M., Di Bartolomeo A. (2025). Pressure-Dependent Current Transport in Vertical BP/MoS2
Heterostructures. Heliyon.

[22] Viscardi L., Durante O., De Stefano S., Intonti K., Kumar A., Pelella A., Giubileo F., Kharsah O., Daniel L., Sleziona S., Schleberger M., Di Bartolomeo A. (2024). Dominant N-Type
Conduction and Fast Photoresponse in BP/MoS2 Heterostructures. Surfaces and Interfaces.

[23] Viscardi L., Mazzotti A., Durante O., Pucher T., Martucciello N., Castellanos-Gomez A., Di Bartolomeo A. (2025). Van Der Waals
WS_2_ /PdSe_2_ Heterostructure as a Visible-Light
Photodetector and Pressure
Optoelectronic Sensor. 2D Mater..

[24] Zeng Y., Hou Z., Yu Z., Huang W., Lv W., Han Q., Zeng T., Luo Y., Lv W., Fang B., Lin Y., Zeng Z., Guo L. (2026). Multimodal Photoelectric Synapses
Based on 2D WS_2_ /PdSe_2_ Heterostructures for
High-Accuracy Neuromorphic Vision. Adv. Funct
Materials.

[25] Liang T., Tian Y., Dai Z., Lenus S., Xie J. (2023). Dual In-Plane/out-of-Plane
Ni2P-BP/MoS2Mott-Schottky Heterostructure for Highly Efficient Hydrogen
Production. J. Alloys Compd..

[26] Li D., Zheng Y., Zhang H., Ye H. (2022). Self-Bending Behavior
and Varying Bending Stiffness of Black Phosphorus/Molybdenum Disulfide
(BP/MoS2) Heterostructure. Nanomaterials.

[27] Li F., Ji S., Wu H., Zhou S., Niu W., Wei L., Bao W., Pu Y. (2020). The Role of the Height Fluctuation Effect in the Tunable
Interfacial Electronic Structure of the Vertically Stacked BP/MoS_2_ Heterojunction. J. Phys. Chem. C.

[28] Wang W., Dong S., Gao Y., Zhang G., Wang K. (2021). Tribological
Behaviours of Black Phosphorus/MoS_2_ Composites as Water-based
Lubrication Additives. Lubrication Science.

[29] Ochedowski O., Marinov K., Scheuschner N., Poloczek A., Bussmann B. K., Maultzsch J., Schleberger M. (2014). Effect of Contaminations and Surface
Preparation on the Work Function of Single Layer MoS_2_. Beilstein J. Nanotechnol..

[30] Xiao J., Zhang Y., Chen H., Xu N., Deng S. (2018). Enhanced Performance
of a Monolayer MoS2/WSe2 Heterojunction as a Photoelectrochemical
Cathode. Nano-Micro Lett..

[31] Viscardi L., Intonti K., Kumar A., Faella E., Pelella A., Giubileo F., Sleziona S., Kharsah O., Schleberger M., Di Bartolomeo A. (2023). Black Phosphorus
Nanosheets in Field Effect Transistors
with Ni and NiCr Contacts. Physica Status Solidi
(b).

[32] Yuan H., Li Z. (2018). Interfacial Properties of Black Phosphorus/Transition Metal Carbide
van Der Waals Heterostructures. Front. Phys..

[33] Di
Bartolomeo A., Intonti K., Peluso L., Di Marco R., Vocca G., Romeo F., Giubileo F., Grillo A., Orhan E. (2025). Metal-Semiconductor Schottky Diode with Landauer’s Formalism. Nano Ex..

[34] Ang Y. S., Yang H. Y., Ang L. K. (2018). Universal Scaling
Laws in Schottky
Heterostructures Based on Two-Dimensional Materials. Phys. Rev. Lett..

[35] Bianchi M. G., Risplendi F., Re Fiorentin M., Cicero G. (2024). Engineering the Electrical
and Optical Properties of WS_2_ Monolayers via Defect Control. Advanced Science.

[36] Kumar S., Kumar A., Kumar A., Chakkar A. G., Betal A., Kumar P., Sahu S., Kumar M. (2024). Catalytic Synergy of
WS_2_ -Anchored PdSe_2_ for Highly Sensitive Hydrogen
Gas Sensor. Nanoscale.

[37] Castellanos-Gomez A., Buscema M., Molenaar R., Singh V., Janssen L., van der Zant H. S. J., Steele G. A. (2014). Deterministic Transfer of Two-Dimensional
Materials by All-Dry Viscoelastic Stamping. 2D Mater..

[38] Di
Bartolomeo A., Pelella A., Liu X., Miao F., Passacantando M., Giubileo F., Grillo A., Iemmo L., Urban F., Liang S. (2019). Pressure-Tunable Ambipolar Conduction
and Hysteresis in Thin Palladium Diselenide Field Effect Transistors. Adv. Funct Materials.

[39] Thakur D., Kumar P., M S., Ramadurai R., Balakrishnan V. (2021). Layer Number Dependent Optical and
Electrical Properties
of CVD Grown Two-Dimensional Anisotropic WS2. Surfaces and Interfaces.

[40] Withanage S. S., Chamlagain B., Johnston A. C., Khondaker S. I. (2021). Charge
Transfer Doping of 2D PdSe_2_ Thin Film and Its Application
in Fabrication of Heterostructures. Adv. Elect
Materials.

[41] Hao D., Chen T., Guo P., Liu D., Wang X., Huang H., Huang J., Shan F., Yang Z. (2023). Artificial
Optoelectronic Synaptic Devices Based on Vertical Organic Field-Effect
Transistors with Low Energy Consumption. Adv.
Compos Hybrid Mater..

[42] Feng S., Li J., Feng L., Liu Z., Wang J., Cui C., Zhou O., Deng L., Xu H., Leng B., Chen X.-Q., Jiang X., Liu B., Zhang X. (2023). Dual-Mode
Conversion of Photodetector and Neuromorphic Vision Sensor via Bias
Voltage Regulation on a Single Device. Adv.
Mater..

[43] Guo H., Guo J., Wang Y., Wang H., Cheng S., Wang Z., Miao Q., Xu X. (2024). An Organic Optoelectronic Synapse
with Multilevel Memory Enabled by Gate Modulation. ACS Appl. Mater. Interfaces.

